# Hidden Markov models identify major movement modes in accelerometer and magnetometer data from four albatross species

**DOI:** 10.1186/s40462-021-00243-z

**Published:** 2021-02-22

**Authors:** Melinda G. Conners, Théo Michelot, Eleanor I. Heywood, Rachael A. Orben, Richard A. Phillips, Alexei L. Vyssotski, Scott A. Shaffer, Lesley H. Thorne

**Affiliations:** 1 School of Marine and Atmospheric Sciences, Stony Brook University, Stony Brook, NY 11794 USA; 2 Centre for Research into Ecological and Environmental Modelling, University of St Andrews, St Andrews, KY169LZ UK; 3 Department of Fisheries and Wildlife, Oregon State University, Hatfield Marine Science Center, 2030 SE Marine Science Dr., Newport, OR 97365 USA; 4 British Antarctic Survey, Natural Environment Research Council, High Cross, Madingley Road, Cambridge, CB3 0ET UK; 5 Institute of Neuroinformatics, University of Zurich and Swiss Federal Institute of Technology (ETH), 8057 Zurich, Switzerland; 6 Department of Biological Sciences, San Jose State University, San Jose, CA 95192-0100 USA

**Keywords:** Accelerometer, Albatross, Animal movement, Behavioral classification, Dynamic soaring, Hidden Markov models, Inertial measurement unit, Magnetometer

## Abstract

**Background:**

Inertial measurement units (IMUs) with high-resolution sensors such as accelerometers are now used extensively to study fine-scale behavior in a wide range of marine and terrestrial animals. Robust and practical methods are required for the computationally-demanding analysis of the resulting large datasets, particularly for automating classification routines that construct behavioral time series and time-activity budgets. Magnetometers are used increasingly to study behavior, but it is not clear how these sensors contribute to the accuracy of behavioral classification methods. Development of effective  classification methodology is key to understanding energetic and life-history implications of foraging and other behaviors.

**Methods:**

We deployed accelerometers and magnetometers on four species of free-ranging albatrosses and evaluated the ability of unsupervised hidden Markov models (HMMs) to identify three major modalities in their behavior: ‘flapping flight’, ‘soaring flight’, and ‘on-water’. The relative contribution of each sensor to classification accuracy was measured by comparing HMM-inferred states with expert classifications identified from stereotypic patterns observed in sensor data.

**Results:**

HMMs provided a flexible and easily interpretable means of classifying behavior from sensor data. Model accuracy was high overall (92%), but varied across behavioral states (87.6, 93.1 and 91.7% for ‘flapping flight’, ‘soaring flight’ and ‘on-water’, respectively). Models built on accelerometer data alone were as accurate as those that also included magnetometer data; however, the latter were useful for investigating slow and periodic behaviors such as dynamic soaring at a fine scale.

**Conclusions:**

The use of IMUs in behavioral studies produces large data sets, necessitating the development of computationally-efficient methods to automate behavioral classification in order to synthesize and interpret underlying patterns. HMMs provide an accessible and robust framework for analyzing complex IMU datasets and comparing behavioral variation among taxa across habitats, time and space.

**Supplementary Information:**

The online version contains supplementary material available at 10.1186/s40462-021-00243-z.

## Background

The rapid development of animal-borne sensors, cameras, and tracking devices has ushered in the current “golden age of bio-logging” [1]. Advancements in biologging technology accelerated our understanding of animal ecology, behavior, and physiology [1–3], with promising applications for conservation management [4]. As biologging devices have become cheaper, smaller and with greater functionality, resulting datasets have become ever-larger and more complex, and there is a critical need for methods that can effectively handle these “big-data” [5, 6]. Indeed, given the proliferation of animal tracking studies, arguably it is the development and implementation of robust, objective methods for data storage, visualization and analysis that limits scientific progress more than data availability per se for many species and systems.

Inertial measurement units (IMUs) with sensors such as triaxial accelerometers, magnetometers, and gyroscopes, are increasingly used in biological studies [7]. Accelerometers are particularly popular tools in studies of animal movement, as they are small, affordable, and battery-efficient [8]. They record instantaneous movement and orientation of the animal body at a high resolution, and are a powerful means of decoding real-time behaviors and their functions in free-ranging animals [9, 10]. The miniaturization of GPS devices greatly improved the resolution of broad-scale movement patterns of animals [11, 12], and concurrent deployments of accelerometers have revealed nuances of fine-scale behavior, such as prey capture events [13, 14], activity-specific energetic costs [15, 16], and even the internal state of animals [17]. Further, the instantaneous nature of accelerometer data can be exploited by behavioral classification methods to derive automated  and objective behavioral time series [18]; these can then be modeled with covariates to assess the drivers of movement and population processes [19].

Tri-axial magnetometers record movement data that are analogous and complementary to that of accelerometers and are being deployed with increasing frequency [20–22]. These sensors record magnetic field orientation and intensity, from which animal heading and angular velocity about the yaw-axis can be derived. Magnetometers are useful for resolving low-acceleration behaviors such as thermal soaring in raptors [23] as well as dynamic behaviors such as running in meerkats (*Suricata suricatta*) [21]. However, despite the purported benefits, behavioral classification routines that incorporate data from both accelerometer and magnetometer sensors are rare, and applied primarily in agricultural and animal welfare studies [24]. Analyzing these data can be challenging because of their complexity and size, the high processing power required, and the frequent lack of in-situ behavioral observations for ground-truthing the patterns observed. Sampling frequencies typically range from 1 to 40 Hz [8], which can quickly lead to extremely high data volumes, particularly when sampling occurs on three axes. Thus, there is a pressing need for more user-friendly methods for analyses of these data.

Hidden Markov models (HMMs) are state-switching time series models [25] and are used increasingly for distinguishing encamped versus directed movement from animal tracking behavior [26]. Used almost exclusively with GPS data, their application to accelerometer data was highlighted recently with promising results [27–29]. Patterson et al. [28] compared the performance of six behavioral classification methods, including HMMs, using taxa-specific metrics such as wingbeat frequency and dive depth derived from accelerometer and pressure data. While they found that all methods performed similarly, they concluded that HMMs provide advantages over other methods given their ability to test the effect of predictor variables on state transition probabilities and because HMMs explicitly model serial autocorrelation [26, 30, 31]. Further, HMMs can incorporate multiple types of data [32, 33], a feature that is highly relevant to biologging studies since IMU devices typically record simultaneous data streams from multiple sensors (e.g. accelerometers, magnetometers, gyroscopes). However, the effectiveness of HMMs for inferring behavior from long time series of multiple high-resolution sensors has not been thoroughly explored in free-ranging animals.

Across taxa and environments, most animal species have evolved locomotory repertoires with multiple movement modes, such as flapping, gliding or soaring in birds; walking, trotting or galloping in terrestrial quadrupeds; and stroking or gliding in marine mammals. Kinematic differences associated with locomotory modes will be reflected in discrete signal patterns recorded by IMU devices, and the larger these differences, the more likely the modes will be captured accurately by behavioral classification methods.

We evaluated the utility of HMMs to classify major movement modes from magnetometer and accelerometer data collected from flying birds using four species of albatross as a case study. Albatrosses are known for their ability to travel vast distances with low energetic costs using a specialized form of flight – dynamic soaring – by exploiting energy from wind and waves [34]. Despite this specialization, albatrosses also use powered flight in the form of flapping when necessary, such as in light wind conditions [35]. Given the large differences in body kinematics associated with flapping and soaring flight, we hypothesized that HMMs would be able to effectively distinguish between these two flight modes as well as identify when birds were sitting on water or land. Our specific objectives were to: 1) classify albatross behavior at sea into three major movement modes: ‘flapping flight’, ‘soaring flight’, and ‘on-water’; 2) determine if the addition of magnetometer data improved classification accuracy; and 3) outline key considerations for using high-resolution sensor data and HMMs to classify major movement modes in animals.

## Methods

### Study species

**Fig. 1:**
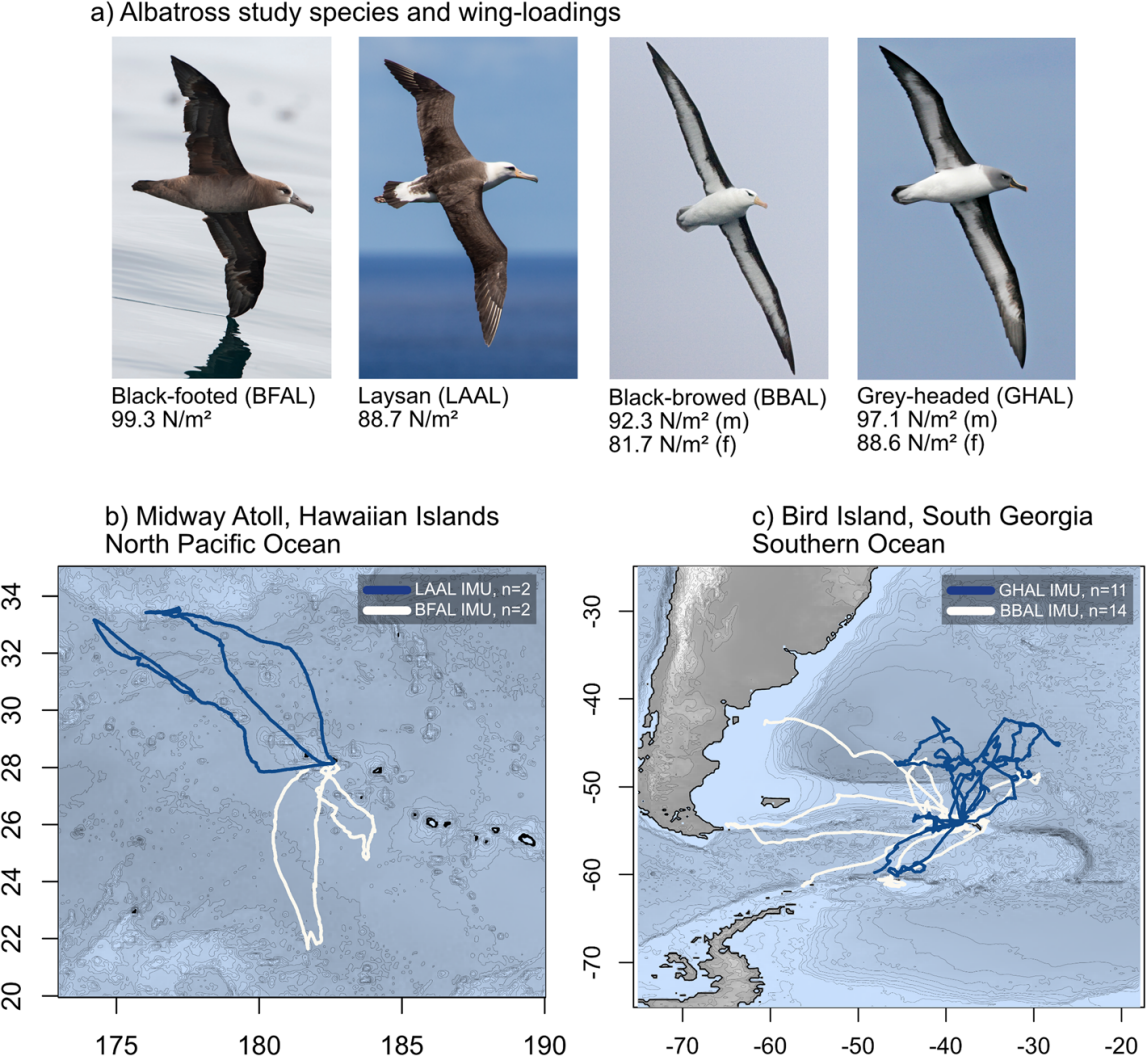
An overview of albatross deployments at the two study sites.**a** Photographs of birds in flight with wing loadings (N/m^2^) for the four study species. GPS tracks of albatrosses simultaneously deployed with IMU devices are displayed from **b** Midway Atoll (Hawaiian Islands) or **c** Bird Island (South Georgia) in the 2018/19 and 2019/20 breeding seasons, respectively

We deployed devices on four albatross species: black-footed (*Phoebastria nigripes*), Laysan (*P. immutabilis*), black-browed (*Thalassarche melanophris*), and grey-headed (*T. chrysostoma*) albatrosses (Fig. 1). All four species are morphologically similar, medium-sized albatrosses that forage in sub-topical to polar waters. Black-footed and black-browed albatrosses are slightly larger-bodied and prefer to forage along shelf-break and shelf-slope waters, whereas Laysan and grey-headed albatrosses tend to forage at oceanic frontal zones [36–38].

### IMU data

**Fig. 2:**
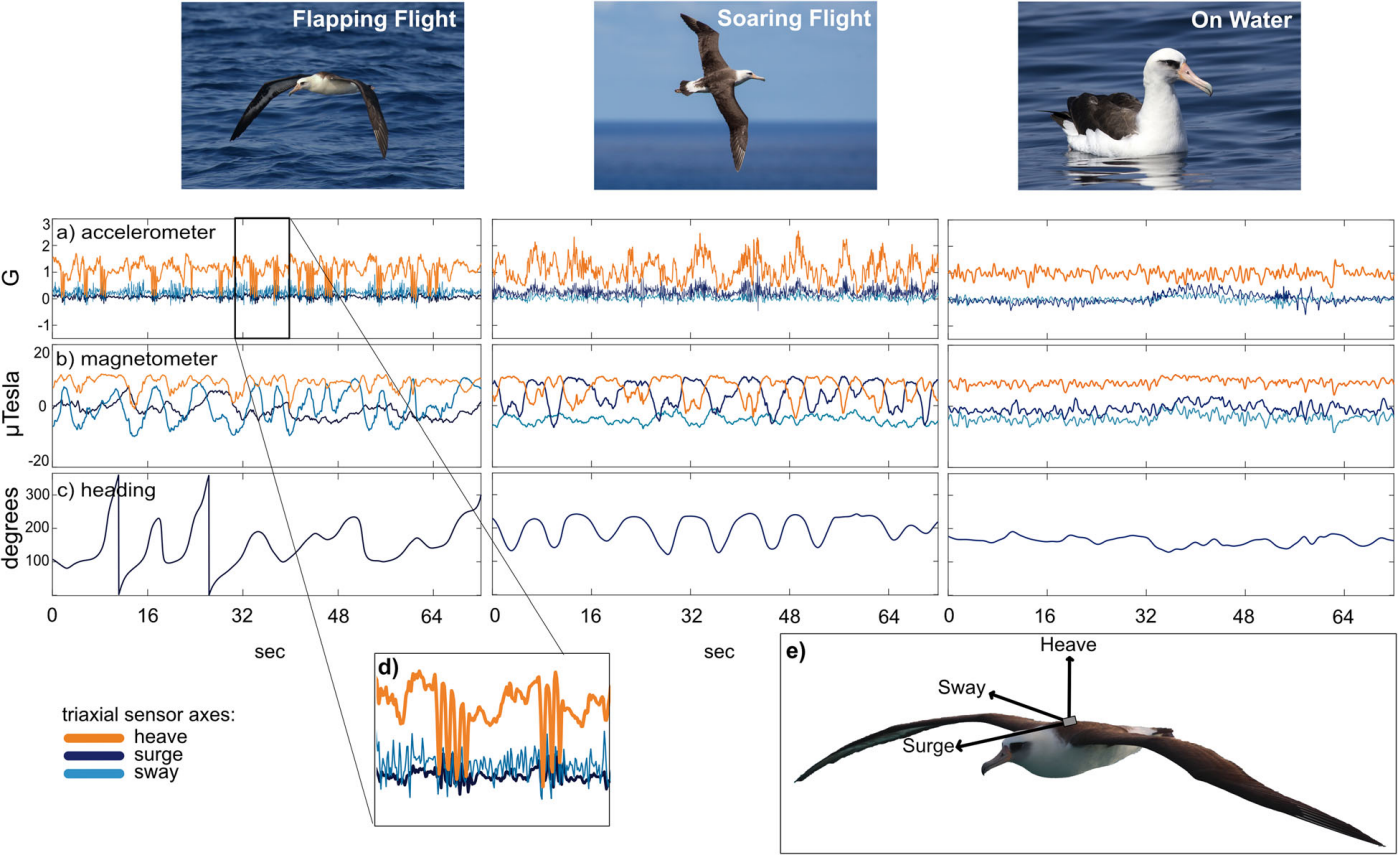
Sensor data representing flapping flight, soaring flight, and ‘on-water’ behavior from a Laysan albatross. Distinct patterns for each behavior are observed in **a** triaxial accelerometer data, **b** triaxial magnetometer data, and **c** heading. **d** A closer look at two isolated bouts of flapping flight. **e** Magnetometer and accelerometer devices were taped centrally onto albatross backs and measured surge acceleration in the anterior-posterior axis, sway acceleration in the medio-lateral axis, and heave acceleration in the dorso-ventral axis

We deployed IMU devices on black-footed and Laysan albatrosses at Midway Atoll National Wildlife Refuge in the North Pacific (28.21 °N, 177.37 °W) during the 2018/19 breeding period, and on black-browed and grey-headed albatrosses during the 2019/20 breeding period on Bird Island, South Georgia in the Southern Ocean (54.00 °S, 38.03 °W) (Table 1). Each bird was equipped with one of two types of device combinations (Table 1): a) Midway Atoll; a GPS tag (Cat-Logger, Perthold Engineering LLC, USA) was paired with a high-resolution IMU (AGM, Technosmart, Italy) containing a 3D accelerometer and 3D magnetometer recording at 25 Hz; or, b) Bird Island; a custom-designed multi-sensor device (Neurologger 2A, Evolocus, New York USA), equipped with an integrated GPS (Cat-Logger), a miniaturized electrocardiogram, and a high-resolution IMU (3D accelerometer, 3D magnetometer) recording at 75 Hz. Tags were attached to central dorsal contour feathers using Tesa tape (#4651, Tesa, Germany) following standard device attachment procedures. IMUs were placed where the *x*, *y*, and *z* axes of the device (the “tag frame”) aligned with the anterior-posterior (surge), medio-lateral (sway), and dorsal-ventral (heave) axes of the birds (the “bird frame”) (Fig. 2e). Tags were deployed for a single foraging trip and removed after a few days to a few weeks depending on foraging trip duration. Total mass of devices was, on average, 1.6, 1.6, 2.1, and 2.0% of the body mass of black-footed, Laysan, black-browed, and grey-headed albatrosses, respectively, and, for all individuals, fell below the 3% recommended percent weight threshold for large-bodied flying seabirds [39].

**Table 1:** Summary of tag deployments on four albatross species at Midway Atoll and Bird Island (South Georgia) in the 2018/19 and 2019/20 breeding seasons, respectively

Albatross Species	IMU Device Type	Range (Acc, Mag)	Resolution (Acc, Mag)	n incubation	n brood-guard	Hours recorded
Black-footed	AGM	8G, 4800 μT	10, 14 bit	0	2	108.2
Laysan	AGM	8G, 4800 μT	10, 14 bit	0	2	140.6
Black-browed	Neurologger 2A	16G, 4900 μT	16, 16 bit	2	12	1363.9
Grey-headed	Neurologger 2A	16G, 4900 μT	16, 16 bit	4	7	1057.2
Total				6	23	2670

### Accelerometer and magnetometer data processing and calibration

All sensor data were pre-processed in MATLAB (2019a) using functions from the Animal Tag Tools Wiki (*http://www.animaltags.or*g), the MATLAB signal processing toolbox, and with customized scripts. Accelerometer and magnetometer data from the Neurologger 2A devices were reduced from 75 Hz to 25 Hz using a decimation function to standardize sampling frequency across tag types. We transformed the sensor frames of the individual accelerometers and magnetometers to align both with each other and with the device frame ([forward, right, up]). Additionally, a frequent, slight tilt in the roll axis of the Neurologger 2A tag frame relative to the bird frame was corrected using a rotation matrix of Euler angles. Roll offsets were identified from accelerometer data when birds were resting on the water where average heave acceleration was assumed to be ~ 1, and average sway and surge acceleration to approximate 0.

Magnetometer datasets were trimmed to remove data at the beginning and end of the deployment where large spikes in the magnetometer data occurred, likely due to the presence of strong local magnetic interference from other tags and field gear. A median filter removed remaining outliers that occurred throughout the time series. Magnetometers are sensitive to hard and soft iron distortions that interfere with detection of the earth’s true magnetic field and require correction. Thus, magnetometer data were calibrated using a data-driven approach (described in Additional File 1). As a second step, each magnetometer channel was rotated according to pitch and roll in a tilt correcting procedure to account for postural offsets [40]. Heading was then calculated as the arctangent of the frame-adjusted *x*- and *y*- magnetometer channels [40]. Heading was converted from radians to degrees in the range of [0–360°] for analysis. Before HMM analyses, we trimmed 2 h from the beginning of sensor datasets to exclude any abnormal behavior related to handling effects.

### Selecting and quantifying movement features from sensor data

Prior to movement feature extraction, we calculated summary time-series metrics from the accelerometer and magnetometer data. A fast Fourier transform (FFT) on total heave acceleration identified dominant frequencies in the signal. Static and dynamic heave, from each axis, were isolated from total acceleration to distinguish postural changes from dynamic movements [10]. Static acceleration was obtained from total acceleration using a 2-s running mean, while dynamic acceleration was calculated by subtracting static acceleration from total acceleration [10, 15]. Overall dynamic body acceleration (ODBA), a commonly used proxy of energy expenditure, was calculated by summing the absolute value of dynamic acceleration across the three axes [15].

Movement features derived from accelerometer and magnetometer data were summarized along the 25 Hz sensor timeseries within 30-s fixed time windows, which was then used as the input for the HMM. We selected 30-s as this was the minimum duration required to capture variability in dynamic soaring arcs captured in heading data. There is no ideal number or set of features across systems and classification methods, and features for HMMs should be selected by careful consideration of those that most effectively distinguish the states of interest [27, 28, 41]. We first summarized the accelerometer and magnetometer data into eight candidate features, based on common techniques (see Fig. 2 in [42]) and a-priori knowledge of flight behavior (see Additional File 2). The eight candidate features are as follows: 1) ‘df’: the dominant frequency (Hz) identified in the FFT on total heave acceleration, 2) ‘hf’: the highest frequency of all dominant frequencies identified in the FFT, 3) ‘ms’: mean static heave acceleration, 4) ‘ss’: standard deviation of static heave acceleration, 5) ‘p5’: the top fifth percentile of static heave acceleration, 6) ‘sh’: circular standard deviation of heading, 7) ‘iqr’: the inter-quantile range of dynamic heave acceleration, and 8) ‘mo’: mean ODBA. To select the final set of features, we first evaluated the degree of correlation among candidate features and identified a smaller set of four features that were minimally correlated to each other: ‘hf’, ‘p5’, ‘mo’, and ‘sh’ (Additional File 2).

### Model selection

**Fig. 3:**
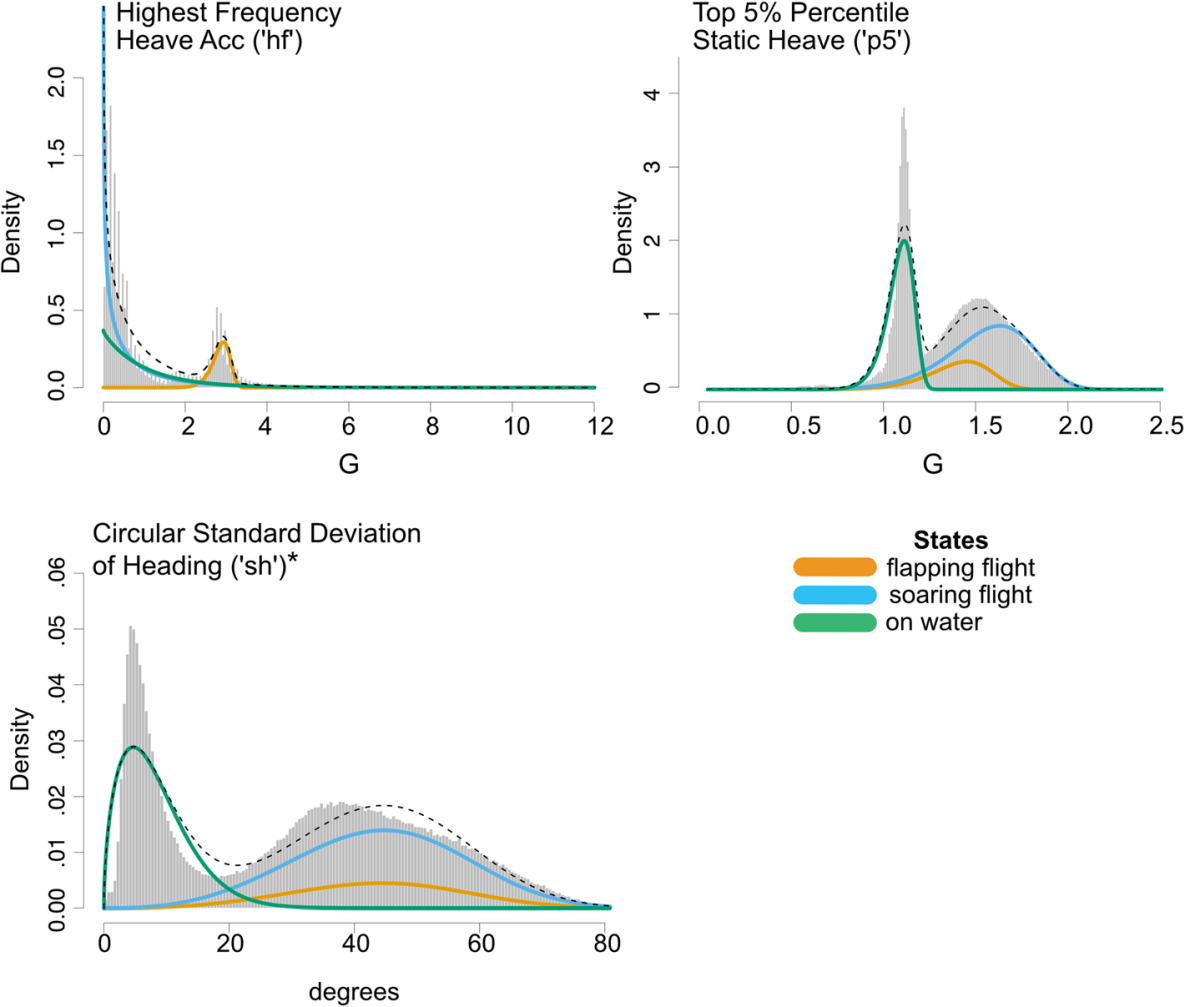
State-dependent density histograms for each input feature (‘hf’, ‘p5’, and ‘sh’). *Circular standard deviation of heading (‘sh’) was only included in Model-1

A series of 3-state HMMs were fitted to the final set of features using the momentuHMM package in R [43]. An initial 3-state model including all four features did not converge on biologically meaningful state distributions, so we ran multiple iterations of HMMs on a subset of data using different combinations of the other three features until state distributions looked appropriate (i.e., until they reflected the patterns one would expect from albatross flight dynamics) (Fig. 3). This resulted in our final set of three features used in all subsequent and final HMM iterations: ‘hf’, ‘p5’, and ‘sh’.

We then ran the model with and without species as a covariate to evaluate its influence on model fit, measured by Aikake information criterion (AIC). Species was included as a fixed effect on transition probabilities only, and not on state distributions since there was high overlap among species in the shape and scale of feature distributions (Additional File 3).

### State classification using hidden Markov models

Since we were interested in understanding the contribution of magnetometer data to the accuracy of behavioral classification, we constructed two final models: 1) Model-1 incorporated all three features, including the magnetometer-derived ‘sh’, and 2) Model-2 excluded ‘sh’ and only used the two accelerometer-derived features ‘hf’ and ‘p5’. Both final models (i.e., Model-1 and Model-2) included species as a fixed effect on transition probabilities.

HMMs were fitted in momentuHMM by numerical maximization of the likelihood, which requires starting values that best estimate the state distributions for each feature [25]. An optimization routine identified the best-fitting model from 25 runs, where each iteration used a randomly generated set of starting values to fit each feature distribution. The best-fitted model from the 25 runs was identified by finding the model with the largest maximum likelihood. We also evaluated the numerical stability of each model as stable models should converge to the same maximum likelihood value in the majority of iterations. Feature distributions were modeled using Weibull probability distributions, specified by single shape and scale parameters. For each feature, a range of starting values were chosen based on the shape and scale of Weibull distributions anticipated for each state. The shape, position, and spread of data distributions of the three features were similar across individuals and we treated datasets from individuals as independent realizations from a common model. Once the models were fitted, we used the Viterbi algorithm to estimate the most likely sequence of states from the fitted model [44]. The 30-s feature dataset contained three short runs of missing data (< 0.02% of the full dataset) but this is not a problem in the HMM framework as parameters are estimated based on the non-missing observations only [26].

### Evaluation of behavioral classification accuracy

To evaluate classification accuracy of Model-1 and Model-2, an expert-driven approach identified our best estimation of “true” states from the sensor data, as we did not have independent observations of bird behavior from video cameras or other methods. While the absence of direct observation precludes the verification of true behavior, previous research has described patterns in sensor data that reflect flapping flight [8, 23, 45], soaring flight [20, 23], and on-water behavior [46] in free-ranging birds, including albatrosses. These patterns are similar across species, are highly distinct from one another (Fig. 2), and were used to manually classify patterns in the sensor data in a validation dataset. While we cannot guarantee that all expert-classified states did indeed capture true behaviors, we are confident in our approach given that we focused on three broad-scale movement patterns that match large differences in signal patterns which are easily discernible by the human eye after familiarization with the data.

The validation dataset used to evaluate classification accuracy was derived from five randomly selected black-browed and grey-headed albatrosses, and each of the two black-footed and Laysan albatrosses. From the sensor data of each validation bird, we selected, randomly, 50 of the 30-s windows used to summarize features for HMM input. These were then assigned, by visually evaluating patterns in the sensor data, into one of three “true” states, giving us a total validation dataset of 700 observations (50 per bird) for comparison with the HMM-inferred states. Observations from periods when the birds were at the colony were removed (*n* = 8). For observations where the sensor data reflected multiple behaviors, we selected the dominant behavior as the “true” state. A confusion matrix was built using the ‘*caret’ package* in R on the resulting 692 observations. Model accuracy was defined as the percentage of observations where HMM-inferred states matched the “true” (expert-classified) states [47, 48].

### Albatross activity budgets

We identified flapping, soaring, and on-water “bouts” as those portions of the HMM-inferred state time series with contiguous observations of each behavioral state. Activity budgets (% time in each state) were constructed from the time series of HMM-inferred states only for at-sea portions of foraging trips in which data were available for all devices (*n* = 18 from the brood-guard) in order to make comparisons across individuals and species.

## Results

IMU devices recorded 2670 h of 3D accelerometer and magnetometer data from 29 individual albatrosses across all species. The trimmed standardized dataset (see *IMU data*) provided a total of 239,564,250 data points summarized into a final dataset of 319,409 observations of each feature as HMM input (Table 1). Devices recorded, on average, for 6.4 ± 2.6 days (mean ± sd) during incubation trips and 3.2 ± 1.6 days during brood-guard, with maxima of just over 8 days before the device memory filled to capacity. On average, incubation trips lasted 9.2 and 7.9 days in duration (measured from co-deployed GPS units) for black-browed and grey-headed albatrosses, respectively. One IMU device recorded the full seven-day duration of an incubation trip, whereas 76% of devices recorded the entire foraging trip during brood-guard (which averaged 4.8, 3.0, 2.4 and 3.0 days for Laysan, black-footed, black-browed and grey-headed albatrosses, respectively).

### Model selection and comparison of behavioral classification accuracy among models

Including a covariate of species as a fixed effect on transition probabilities slightly improved model fit as reflected in a marginally lower AIC (2,600,123 vs 2,601,474). All final model iterations converged on state-dependent parameters (Fig. 3, Additional File 4) and displayed numerical stability by settling on the same likelihood for the majority of the iterations.

**Fig. 4:**
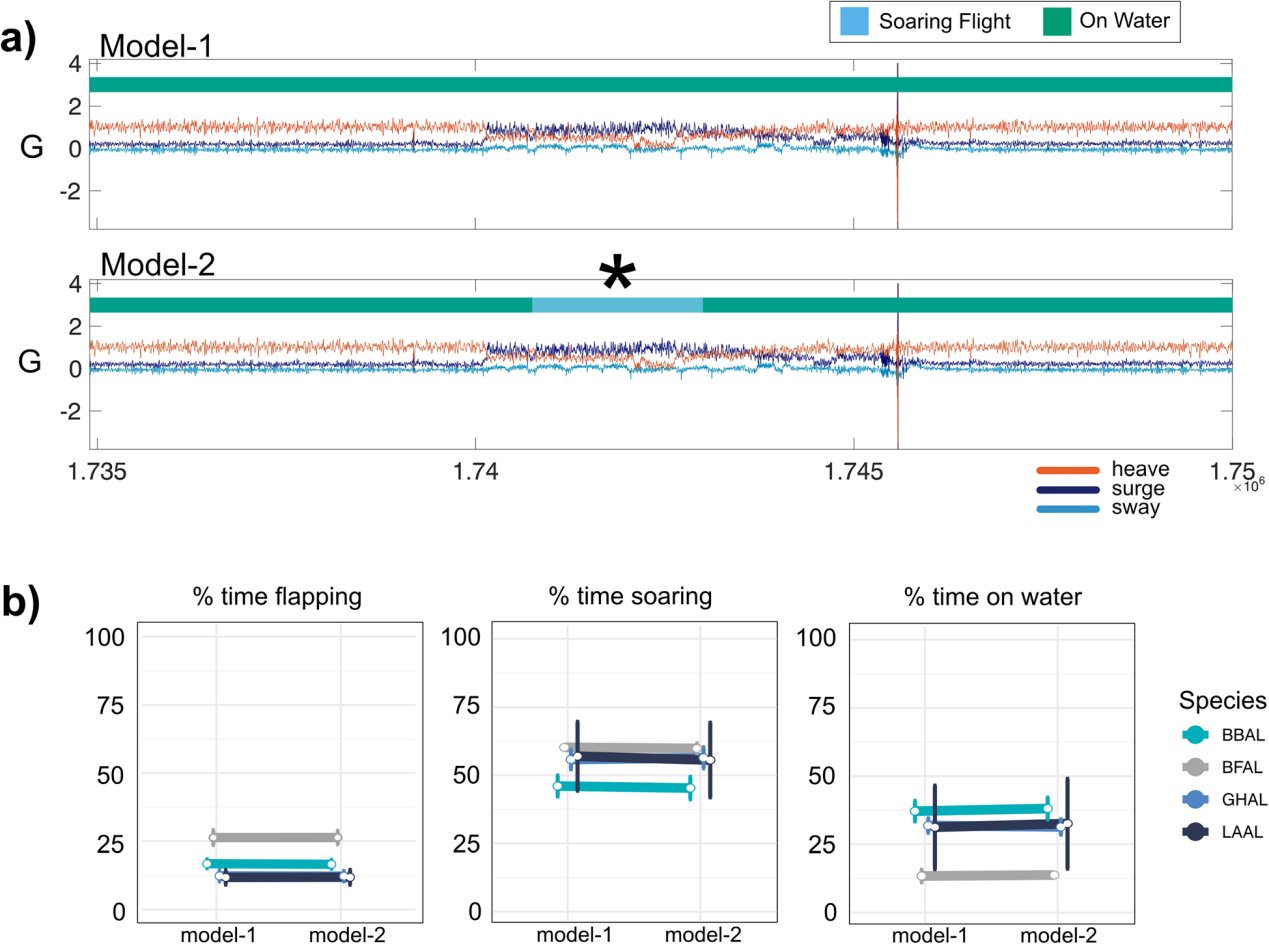
Comparison of classification accuracy between model-1 and model-2. **a** An 8-min segment of triaxial accelerometer data from a grey-headed albatross demonstrates a state misclassification common in Model-2, where ‘on-water’ behavior (green) was misclassified as soaring flight (blue). **b** Across species, differences in behavioral budgets derived from HMM-inferred states between Model-1 and Model-2 were negligible

Both Model-1 and Model-2 showed high and nearly equal classification accuracy (91.9 and 91.5%, respectively, Table 2). Both models also performed well across species with all species having accuracies > 90%, with the exception of black-footed albatrosses which were modeled with slightly lower accuracies of 84 and 86% in Model-1 and Model-2, respectively (Table 3). State-specific accuracy was highest for soaring flight (93% in both models) and lowest for flapping flight (87%). Accuracy for flapping flight was comparatively low in both Laysan and grey-headed albatrosses (71.4 and 76.7%, respectively). Low classification accuracy for flapping flight in these two species reduced the global-level accuracy for that behavioral state, as classification accuracy for flapping flight was much higher for the two other species. Misclassifications of flapping flight were primarily soaring flight, though in Model-2, there was an increase in flapping flight being misclassified as ‘on-water’ behavior relative to Model-1 (Fig. 4a). ‘On-water’ misclassifications were uniformly confused as soaring flight by both models (Table 3). State-transition probabilities indicated that the most persistent state was the ‘on-water’ behavior followed by ‘soaring flight’ (Table 4).

**Table 2:** Transition probability matrices for HMMs showing the probability of transitioning from each state at time t to time t + 1. Transition probabilities are displayed as estimates with 95% confidence intervals in parentheses

**Model-1: Accelerometer and Magnetometer**			
	STATE AT TIME t + 1		
STATE AT TIME t	Flapping Flight	Soaring Flight	On-Water
Flapping Flight	0.778 (0.772, 0.783)	0.204 (0.199, 0.209)	0.019 (0.017, 0.020)
Soaring Flight	0.083 (0.080, 0.085)	0.900 (0.898, 0.903)	0.017 (0.016, 0.018)
On-Water	0.010 (0.009, 0.011)	0.016 (0.015, 0.018)	0.974 (0.017, 0.020)
**Model-2: Accelerometer only**			
	STATE AT TIME t + 1		
STATE AT TIME t	Flapping Flight	Soaring Flight	On-Water
Flapping Flight	0.781 (0.776, 0.786)	0.194 (0.189, 0.199)	0.025 (0.023, 0.027)
Soaring Flight	0.080 (0.078, 0.082)	0.911 (0.908, 0.913)	0.009 (0.008, 0.010)
On-Water	0.011 (0.010, 0.012)	0.008 (0.007, 0.009)	0.980 (0.979, 0.981)

**Table 3:** Confusion matrices depicting model classification accuracy as the percentage of correct behavioral assignments for each behavior. Overall classification accuracies are displayed as estimates with 95% confidence intervals in parentheses

**Model-1: Accelerometer and Magnetometer**			
	*Visually-assigned*		
*HMM-assigned*	Flapping Flight	Soaring Flight	On-Water
Flapping Flight	**87.6**	6.9	0.9
Soaring Flight	12.4	**93.1**	7.4
On-Water	0	0	**91.7**
**Overall accuracy: 91.9% (89.6, 93.8)**			
**Model-2: Accelerometer only**			
	*Visually-assigned*		
*HMM-assigned*	Flapping Flight	Soaring Flight	On-Water
Flapping Flight	**86.6**	7.1	0.5
Soaring Flight	10.3	**92.6**	7.9
On-Water	3.1	0.3	**91.7**
**Overall accuracy: 91.5% (89.1, 93.5)**

**Table 4:** Species-specific confusion matrices depicting classification accuracy from Model-1 as the percentage of correct behavioral assignments for each behavior. Overall classification accuracies are displayed as estimates with 95% confidence intervals in parentheses

**Black-footed albatross** (*n* = 100)			
	*Visually-assigned*		
*HMM-assigned*	Flapping Flight	Soaring Flight	On-Water
Flapping Flight	**92.0**	18.7	0
Soaring Flight	8.0	**81.3**	18.2
On-Water	0	0	**81.8**
	**Overall accuracy: 84.0** (**75.3, 90.6)**		
**Laysan albatross** (*n* = 100)			
	*Visually-assigned*		
*HMM-assigned*	Flapping Flight	Soaring Flight	On-Water
Flapping Flight	**71.4**	1.7	0
Soaring Flight	28.6	**98.3**	6.1
On-Water	0	0	**93.9**
	**Overall accuracy: 95.0 (88.7, 98.4)**		
**Black-browed albatross** (*n* = 242)			
	*Visually-assigned*		
*HMM-assigned*	Flapping Flight	Soaring Flight	On-Water
Flapping Flight	**94.9**	3.6	2.1
Soaring Flight	5.1	**96.4**	6.5
On-Water	0	0	**91.4**
	**Overall accuracy: 94.2% (90.5, 96.8)**		
**Grey-headed albatross** (*n* = 250)			
	*Visually-assigned*		
*HMM-assigned*	Flapping Flight	Soaring Flight	On-Water
Flapping Flight	**76.9**	6.2	0
Soaring Flight	23.1	**93.8**	7.6
On-Water	0	0	**92.4**
	**Overall accuracy: 91.6 (87.5, 94.7)**		

### Albatross activity budgets

**Fig. 5:**
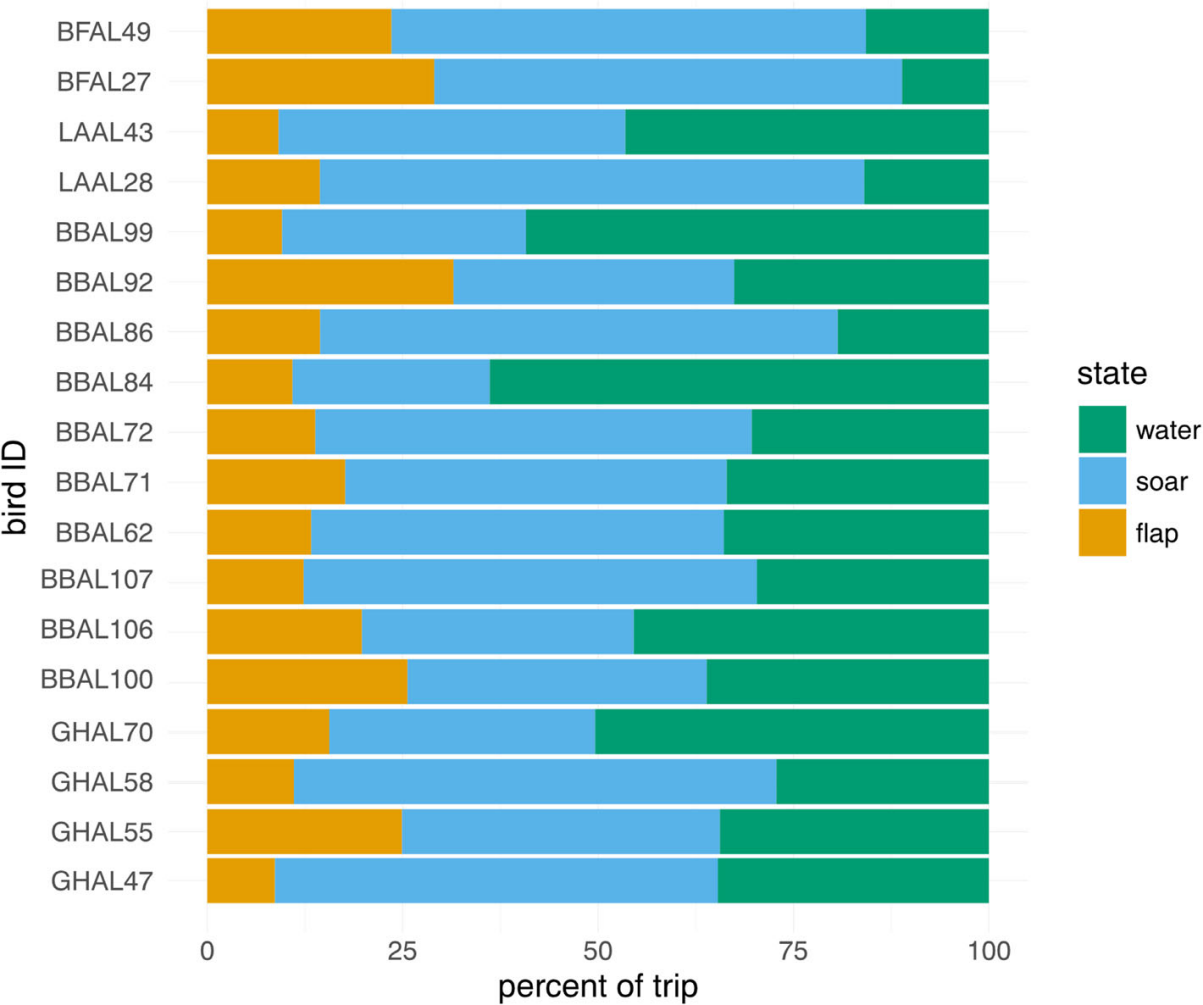
Individual activity budgets during foraging trips of four species of albatrosses tracked during the brood-guard period, constructed from HMM-inferred states (Model-1)

Albatrosses foraging in the brood-guard phase typically spent the least amount of their trip (17.0% on average) in energetically costly flapping flight, though this varied among individuals from 8.7% (in a grey-headed albatross) to 31.5% in a (black-browed albatross; Fig. 5). When in flight, birds spent on average 26.2% of flight time flapping, though this varied greatly among individuals (from 13.3% of flight time in a grey-headed albatross to 46.7% of flight time in a black-browed albatross). Time spent ‘on-water’ ranged from 11.1% in a black-footed albatross to 63.9% in a black-browed albatross. Contiguous flapping flight bouts had the shortest durations of all states, lasting on average for 1.9 min, although one black-browed albatross engaged in a flapping bout for 101 min. Soaring bouts lasted on average for 5.5 min, though one grey-headed albatross had a soaring bout that persisted for 5.3 h. ‘On-water’ bouts were the most persistent behavioral bouts, on average lasting for 24.4 min, though one black-browed albatross spent nearly 9 h continuously on the water.

The slight decrease in accuracy of state classification from Model-1 to Model-2 had a negligible impact on species-level activity budgets (Fig. 4b), though changes in activity budgets were more apparent in some individuals compared to others. For example, the percent time spent soaring and on the water for most individuals changed ≤2% when comparing activity budgets constructed from Model-1 versus Model-2; however, for one individual, time spent ‘on-water ‘increased by 7.5% while time spent soaring decreased by 7.0%. Time spent in flapping flight changed < 1% for all individuals between models.

## Discussion

### HMMs effectively distinguish major movement modes in albatross

Using data from four species of albatrosses, we demonstrated that HMMs can provide a robust and objective means for classifying major movement modes in free-ranging animals using multi-sensor data from high resolution IMUs. An HMM built on features derived from triaxial accelerometer and magnetometer data streams successfully identified flight mode or ‘on-water’ behavior with high accuracy (92%). High classification accuracy across species highlights the flexibility of a single HMM to distinguish movement modes across functionally and morphologically similar species. The efficacy of a single model reflects that the large differences seen in signal characteristics of the three major movement modes were likely larger than the variation among and within species.

### Opportunities and limitations in interpreting state assignments from HMMs

Inferring states with HMMs resulted in a fine-scale behavioral time series for free-ranging albatrosses. Our modelling framework targeted three major movement modes – flapping flight, soaring flight, and ‘on-water’ – the proportions of which are adjusted by foraging birds according to environmental conditions and energetic trade-offs [49, 50]. Albatrosses recognizably have larger behavioral repertoires than the three states targeted in our study [51], with increasingly fine-scale behaviors nested in a hierarchical fashion. There are multiple approaches for increasing the resolution of behaviors using an HMM framework: a more complicated 4-, 5-, or even 6-state model could fit increasingly nuanced behaviors, for example distinguishing resting from sit-and-wait foraging, which is used frequently by black-footed and grey-headed albatrosses [12, 52] and accounts for 35% of prey consumed by grey-headed albatrosses [52]. Alternatively, one could apply a hierarchical HMMs to classify behavioral states occurring at different time scales [32, 33].

However, HMMs can become increasingly complex, computationally demanding, and difficult to interpret as states, features, and hierarchical levels are added, particularly in an unsupervised modeling framework [53]. Alternative analyses that occur after state classification may facilitate the identification of additional, biologically relevant states without requiring a more complex (and computationally time-consuming) modelling framework. Supplementary layers of data could be used with the time-series of HMM-inferred states in a decision-tree analysis to further categorize broad behavioral classes into increasingly nuanced subclasses. For example, an ODBA-based activity-level threshold could further classify the HMM-inferred ‘on-water’ state as active or passive (i.e. resting) depending on whether ODBA is above or below a threshold value. Similarly, a time series of pitch from accelerometer data could be used to identify diving behavior within ‘on-water’ bouts to better identify this type of active foraging, which is important for multiple albatross species [12, 52, 54]. Diving in albatrosses is otherwise very hard to distinguish even with concurrent data on pressure from time-depth recorders (TDRs) and immersion loggers [55].

### Using magnetometers to decode animal behavior: some practical recommendations

We evaluated the contribution of triaxial magnetometer data, in addition to triaxial accelerometer data, for classifying major movement modes of albatrosses. Including the magnetometer-derived feature in the HMM did not meaningfully improve classification accuracy (Table 3). While classification accuracy was similar, Model-2 (the accelerometer only model) more frequently misclassified actual ‘on-water’ behavior as flight behavior, though these misclassifications occurred at low rates (Table 3, Fig. 4a). Despite these slight differences in state assignment, behavioral time budgets derived from the two models were nearly identical (Fig. 4b). This demonstrates that accelerometer data alone can be sufficient for behavioral classification routines focusing on broad movement classes, even in species that predominantly soar.

**Fig. 6:**
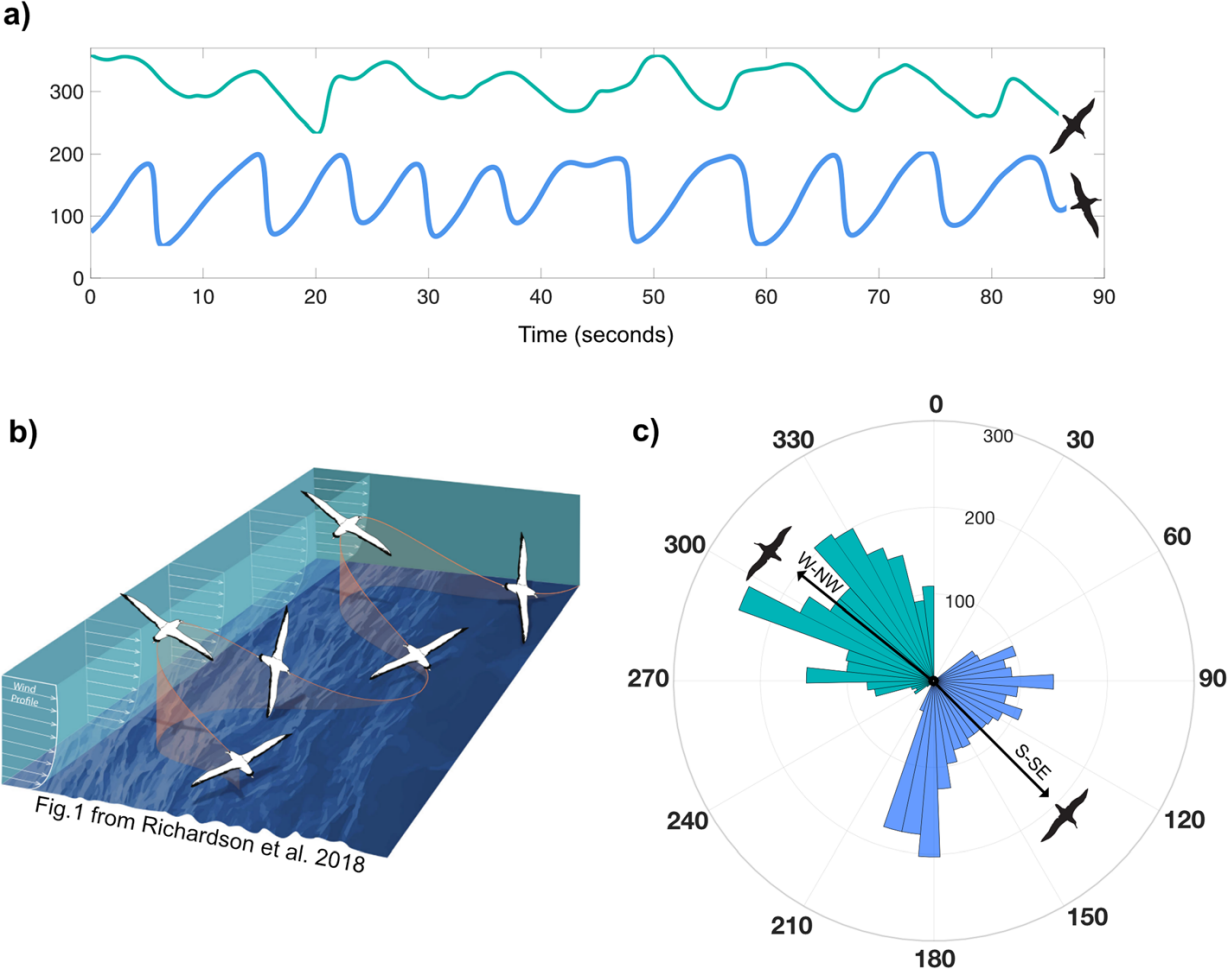
Heading derived from triaxial accelerometer and magnetometer data streams used to characterize dynamic soaring. **a** Two segments of heading (in degrees) from different times along a black-browed albatross trip demonstrate varying patterns in soaring flight. **b** A schematic from Richardson et al., 2018 demonstrates the periodic nature of dynamic soaring. **c** A polar histogram of the heading data used in panel (**a**)

Though we did not find magnetometers improved classification of major movement modes in albatrosses, these sensors and others deployed in addition to accelerometers have improved classification accuracy in other systems [24]. Further, compared to accelerometers, magnetometers are much more effective at characterizing behaviors that involve rotation around the yaw axis (i.e. heading changes) or that manifest primarily as slow, periodic changes in orientation [20]. For example, patterns of heading or angular velocity derived from magnetometers could be used to describe how albatrosses adjust soaring flight within their trip (Fig. 6). The frequency and amplitude of cycles that characterize dynamic soaring [56] could reveal how birds adjust soaring when responding to environmental cues, such as wind variability. Novel metrics derived from magnetometer data, such as the recently described AVeY (angular rotation about the yaw axis) may prove to be equivalent, or better, proxies of energy expenditure for soaring birds than traditional accelerometer-derived metrics such as ODBA [22], given the energetic cost associated with body rotations [57].

The benefits of including a magnetometer must be balanced against both the increased load on the study animal (larger power demand, requiring a bigger battery) and the analytical cost (larger data volume, increased pre-processing time, need for calibration). For species that can only carry small loads, our results suggest that accelerometers alone may be sufficient for classifying broad movement modes using HMMs. Other classification studies have also highlighted the utility of accelerometer data over that from other sensors [58]. Nonetheless, for larger species, and particularly for those with behaviors that occur in a slow, periodic fashion, it may be beneficial to co-deploy magnetometers with accelerometers on an initial subset of animals to explore their unique contribution in terms of behavioral information. It is worth noting, too, that heading can be obtained from GPS devices sampling at high frequency (e.g., 1 Hz, 1 s), and indeed these have been deployed on albatrosses to describe dynamic soaring [59, 60]. However, the battery consumption of GPS devices is much greater than that of magnetometers, limiting recording duration at very high temporal resolution, particularly given the maximum mass of devices that could be deployed on smaller species without deleterious effects.

### Ecological insights

We demonstrated the efficacy of the HMM modeling framework for classifying major movement modes in albatrosses from high-resolution sensor data. While our study was limited to four closely related seabird species, the basic framework should apply across taxa and environmental contexts as long as the targeted behaviors involve major differences in body kinematics. Generalized patterns in movement and locomotion exist across diverse taxa, shaped by common principles such as optimal foraging theory and physiological principles that increase energetic efficiency [61, 62]. Similar kinematics of locomotion across species and taxa support the potential for behavioral classification models that are built in one system to be applied in others, with minor adjustments. For example, wandering albatrosses (*Diomedea exulans*) are 2-3x the mass and have wing-loadings that are 1.6–1.8x greater than the species in our study [63] but their frequency of flapping flight (measured in heave acceleration) is, on average, 2.5–2.7 Hz [64] – the same as in the present study (‘hf’ in Fig. 3) and in an additional study of black-browed albatrosses [45]. Similarly, two studies found that humans, despite large variability in height and leg lengths, had a preferred walking frequency of ~ 1.77 Hz [65, 66]. Further, Gleiss et al. [62] highlighted similar acceleration patterns of propulsive movements in species as disparate as southern elephant seals (*Mirounga leonina*), whale sharks (*Rhincodon typus*), and European starlings (*Sturnus vulgaris*). Applying this concept, machine-learning methods developed with accelerometer data from domestic dogs *Canis lupis familiarus* have been successfully used to predict behavior in morphologically-similar wild species such as wolves *C. lupus* [67] and cheetahs *Acinonyx jubatus* [68].

That a single HMM in our study effectively captured broad movement patterns from IMU data across similar species highlights the potential of this method to disentangle high-resolution behavioral data from free-ranging animals that are difficult to observe. For many free-ranging species, and particularly for those that are wide-ranging like many marine species, classifying behavior will require an unsupervised approach since obtaining simultaneous direct observations under a range of environmental conditions is difficult if not impossible. That animals adhere, then, to movement patterns conserved across taxa bodes well for the construction and interpretation of unsupervised movement models from high resolution sensor data without access to the same level of direct observations that are seen in HMMs applied to IMU data from other fields, such as in agricultural research. Indeed, the majority of studies applying HMMs to high-resolution IMU sensor data involve models built in a supervised fashion for domesticated animals in which behavior can be readily and simultaneously observed (e.g., [69, 70]). While there is some opportunity for collecting direct behavioral observations from free-ranging animals using animal-borne cameras, this is primarily limited to larger species and recordings will be limited in duration due to the high-power demand of video recordings.

Thus, the vast majority of IMU sensor data will come from free-ranging animals without direct observations. This, however, does not prohibit all forms of validation, since behavior can also be inferred without direct observation. In our study it was possible to identify general patterns in the sensor data (e.g., Fig. 2) to deduce the most likely broad-scale behavior that those patterns represent, based on extensive knowledge of the flight styles of our study species, an understanding of how activity and orientation is reflected in patterns from IMU sensors, and previous research on sensor patterns associated with movement modes in similar species. While this is not equivalent to direct observations that can ground truth classification validations, it will likely have to suffice for most studies until cameras decrease in size and increase in recording duration. The ability to interpret behavior from sensor data in wild animals without matching direct observations will in most cases be limited to broad movement classes that are more easily interpreted from sensor data, as in our study. Conversely, nuanced behaviors or those that are highly variable among individuals and species (e.g., prey capture, bathing, socializing) will require extensive ground-truthing with direct observations (e.g. video [71]) or additional sensors (e.g., acoustic recorders [72]).

### Key considerations when using HMMs to classify major movement modes in animals

HMMs have rapidly gained traction in ecology as a modeling framework because they can effectively handle time series with complex structures and because of the growing availability of user-friendly and open-source software and tutorials for their implementation [31, 43, 73]. Fast algorithms have been developed for fitting HMMs and estimating hidden states, making them ideally suited to analyze large data sets obtained from IMU sensors [73]. Further, HMMs are equipped to deal with missing data on an otherwise regular grid [25, 26], which can be common in animal movement studies, although the extent of missing data should be negligible [25]. If gaps in data are large enough, one would want to either interpolate the data (if that is appropriate for the type and size of the missing data), or, treat the contiguous data segments as separate time series (e.g. [74]).

Using HMMs to classify behavior from high-resolution sensor data requires considerable knowledge of the study system to inform the specification of an effective modelling structure [53, 73]. Fortunately, many thorough and comprehensive reviews exist in the literature on best practices for implementing HMMs in ecology and animal movement in general [53, 73] and on HMMs in ecology using accelerometer data [27]. Below, we briefly outline some targeted considerations and recommendations informed by our specific experience inferring behavioral states from high-resolution data on albatrosses using the HMM framework:

#### Unsupervised or supervised

HMMs can be constructed in one of two frameworks: unsupervised or supervised (or semi-supervised), and the applications of each for classifying accelerometer data are described in full in Leos-Barajas et al. [27]. In brief, unsupervised models are fundamentally data-driven for determining state-dependent distributions, while supervised (or semi-supervised) are informed by labeled data. The inclusion of pre-classified data can greatly help with the identification and interpretation of the hidden states, but it requires obtaining direct observations of the tagged animals, or manually assigning states to observations based on prior knowledge. This procedure is often prohibitively costly, and most animal movement studies follow the unsupervised approach.

Our approach was to use an unsupervised modeling framework as no paired data from direct observations were available. However, even in unsupervised frameworks, the HMM modeling framework requires some a priori information on the targeted behavioral states. For example, HMMs require the user to define state-dependent distribution classes (i.e. gamma, von Mises, Weibull) as well as to provide starting values, informed by anticipated characteristics of state-dependent distributions, that, if selected well, will reduce the chance the model will converge on local, rather than global, maxima [73]. In principle the choice of these initial parameters shouldn’t have any effect on the estimated state-dependent distributions; however, in practice they often do, because the estimation can easily run into numerical problems if the initial parameters are chosen in an uninformed manner. This underscores how critical it is to have sound kinematic knowledge about the species that is being modeled in the HMM framework, not only for model interpretation, but also for model construction even in an unsupervised fashion.

#### Defining model performance

There are several approaches for describing the performance of an HMM to capture features of the data, including (1) goodness-of-fit, which reflects how well the mixture of observation distributions fits the data, and is often quantified using pseudo-residuals (described in Ch. 6 of Zucchini et al. [25]), and (2) evaluating classification accuracy by comparing HMM-inferred states with a subset of data manually labelled into “true” behavioral categories. Typically, checking the normality and autocorrelation of pseudo-residuals is the primary means of evaluating model performance for unsupervised HMMs since there is no data to ground-truth the classification. These goodness-of-fit measures do not assess how well the estimated states match the expected behaviors, however, and classification accuracy may be a better metric of performance for studies focusing on state classification and interpretation [53]. In addition, standard model selection criteria (such as AIC) tend to favor models with many states, which describe the data well at the expense of interpretability [53]. Inspecting the pseudo-residuals to ensure a reasonable fit is recommended [73], but when the primary objective is to classify a targeted set of behaviors, the focus of model checking and model selection should be to identify the model with state-dependent distributions that are most biologically interpretable [53, 73].

#### Using HMMs in multi-species studies

For classification studies involving multiple species, a single HMM may suffice if the targeted movement patterns are kinematically distinct and the inter-specific differences in signal patterns are small. Incorporating individuals from multiple species in a single model has many benefits, including the simplicity of running one model versus many, the buffering of small sample sizes for individual species by pooling data (e.g., black-footed and Laysan albatrosses in this study), and potentially increasing the capability to enhance model complexity and improve state inference [75, 76]. The degree of similarity in movement patterns among species should be explored early in analysis by visualizing and comparing histograms of the derived features (e.g., Additional File 3). However, while histograms may be informative in many situations, they might not always be sufficient for determining the best approach; as such, fitting multiple preliminarily models on a subset of data will likely be helpful. The HMM framework is equipped to include covariates, and species can be included as a fixed effect on both state transition probabilities (as in this study) and state-dependent distribution parameters. Given the high overlap in feature histograms among species, we did not include species as a fixed effect on state-dependent distribution parameters, though given the lower accuracy of Laysan and grey-headed albatrosses in the single model, adjusting the HMM in this manner may improve classification accuracy across species. Ultimately, if classification accuracy from a single model is low or highly variable across species, even after the inclusion of a species covariate, individual HMMs fitted to each species may be worth exploring if sample size permits.

#### Feature selection

The selection of a small number of appropriate features as input to the HMM is critical for maximizing classification accuracy [77]. Studies using accelerometer data to classify animal behavior often use a large number of input features (often > 10, and up to 152, reviewed in Patterson et al. [28]). However, additional features added to the unsupervised HMM modeling framework rapidly increases model complexity and computational demands [73]. As described above, each additional feature requires some a priori knowledge about the anticipated state-dependent distributions in order to provide sensible starting values and selecting appropriate starting values for many features can quickly become prohibitively complex. Futher, recent studies have demonstrated little to no gain in accuracy from additional features beyond two to four [28, 58, 78]. Identification of candidate features from those that are commonly used [42] should be informed by knowledge of animal behavior (e.g., it would make biological sense to target metrics derived from heave acceleration for a fluking dolphin versus sway acceleration for a swimming fish) and from tag position on the animal which affects signal patterns. An initial larger set of candidate features can then be narrowed down to an optimal set (the smallest set that most effectively distinguishes behaviors) by either a manual inspection of histograms and correlation matrices, as in this study, or through a formal forward selection procedure, (e.g. [78]). If there is minimal knowledge of the behavior of the targeted species to help narrow down candidate features, alternative classification methods, such as ensemble classifiers like random forest models that effectively handle large sets of features, may be a better approach.

The time window chosen to summarize features from raw sensor data will also have a major influence on classification accuracy. Since behaviors often operate on different time scales, it can be difficult if not impossible to identify a single window that effectively captures all targeted behaviors. In order to properly quantify signal patterns in distinct behaviors, time windows should not be smaller than the typical time for a “unit” of behavior to occur (e.g., one arc in a dynamic soaring cycle, or one ‘flap’ for flapping behavior), Further, fixed time windows will inevitably be problematic if behavioral transitions can occur therein. Sliding windows and overlapping windows can resolve some of these issues [79]; however, for high-resolution sensor data, this approach results in feature datasets of a similar size to the raw sensor data. For datasets like ours that contain hundreds of millions of rows, this would be prohibitively large to run in an HMM context. Thus, finding the optimal time window may require some experimentation in an initial set of preliminary HMMs run on a subset of data.

#### Selecting the number of states

A primary consideration in using an HMM as a behavioral classification procedure is to avoid overfitting a model with states that are not biologically meaningful. As described earlier, model selection criteria tend to favor HMMs with superfluous states that reflect unaccounted-for structure in the data, rather than true biological states, and should be used with care, particularly when the objective of the HMM is to classify states with certain behaviors in mind [53]. Pohle et al. [53] recommend running a few initial HMMs with varying numbers of states (< 4) to develop an understanding of how the models identify key patterns, and then evaluate these, using pseudo-residuals and biological intuition.

## Conclusion

Overall, we demonstrate that accelerometer and magnetometer sensors, paired with HMMs, offer a promising set of tools that can be applied to a wide range of species and questions for an improved understanding of animal behavior and energetics. We constructed a framework that used a single HMM to predict with 92% accuracy, the major movement modes of four albatross species. Magnetometer did not meaningfully improve HMM classification accuracy but is potentially of much greater value for quantifying fine-scale details of other behaviors, such as dynamic soaring and associated energetic proxies.

## Supplementary Information

**Additional file 1: Supplemental Information and Figure S1 and Table S1. **Details of magnetometer calibration.

**Additional file 2: Supplemental Table S2.** Description of candidation features for the hidden Markov models.

**Additional file 3: Supplemental Figure S2.** Feature distributions across species.

**Additional file 4: Supplemental Table S3.** HMM-converged parameters for the Weibull distribution of each feature and state.

## Data Availability

Scripts and a subset of data used in analysis can be found on Github (github.com/melindaconners/HMMalbatross/). Raw accelerometer and magnetometer datasets used in the study are available from the corresponding author at reasonable request.

## Abbreviations

BFAL: Black-footed albatross
LAAL: Laysan albatross
BBAL: Black-browed albatross
GHAL: Grey-headed albatross
ODBA: Overall Dynamic Body Acceleration
HMM: Hidden Markov Model
IMU: Inertial Measurement Unit
AVeY: Angular velocity about the yaw axis
FFT: Fast Fourier transform
