# Supplementary material for: Hidden Markov models identify major movement modes in accelerometer and magnetometer data from four albatross species
Source: Mov Ecol. 2021 Feb 22;9:7. doi: 10.1186/s40462-021-00243-z (PMC7901071; doi:10.1186/s40462-021-00243-z)
Supplement: Supplementary file 1 — Additional file 1: Supplemental Information and Figure S1 and Table S1. Details of magnetometer calibration. [file 40462_2021_243_MOESM1_ESM.docx]

Additional File 1

**Magnetometer calibration**

Hard-iron distortions are caused by ferrous materials that have their own permanent magnetic field which shift the magnetometer’s reading of the magnetic field by a constant offset from the origin. Soft iron distortion, on the other hand, occurs when non-magnetic ferrous material in the device cause the earth’s magnetic field to flow preferentially through it. Both of these distortions can be visualized in the triaxial magnetic field intensity scatter plot: with no distortion, the magnetometer data should form a perfect sphere with a center of [0,0,0] in Cartesian coordinate space; hard-iron distortion will shift the position of the sphere away from the true origin while soft-iron distortion will cause the sphere to morph into an ellipsoid form. Thus, correcting for both hard and soft iron distortions requires using an ellipsoid-fitting algorithm to reshape the ellipsoid into a uniform sphere and a correction factor to shift the center of the sphere back to the origin.

Traditionally, these correction factors are calculated using a bench calibration dataset typically recorded on the magnetometer device before each deployment. During these calibration recordings, the device is moved in a series of 360° rolls around each axis so that the tag experiences the full spherical range of the magnetic field. However, in addition to ferrous materials, magnetometers are influenced by temperature and these effects may be difficult to model in bench calibrations, particularly in wide-ranging animals like albatross that encounter dynamic temperature ranges as they cross different habitats or move from air to water. Data-driven calibration methods fit an ellipsoid to the full magnetometer dataset from an animal deployment, rather than from an initial user-defined set of movements of a bench calibration, and they may provide a more robust calibration for long deployments (Johnson, 2011). Calibrations performed using the data-driven approach require magnetometer data from three axes and from animals that move dynamically enough that the resulting magnetometer data covers a sufficient amount of the field intensity sphere. Albatrosses fly predominately using dynamic soaring, in which they change their body orientation to the wind, and an initial inspection of magnetometer data from our deployments indicated sufficient spherical coverage for the data-driven approach (e.g. Fig S1). To calibrate the data, we applied an ellipsoid fit algorithm to each trimmed and median-filtered triaxial magnetometer dataset and used the ellipsoid fit correction factors to reshape the data. The data were subsequently shifted back to the origin and scaled to a radius of one. Each calibration was evaluated by calculating the residual error of the calibrated sphere from a uniform sphere centered on the origin, and we accepted calibrations if the residual error was less than or equal to 10% (Table S1). When the residual error of magnetometer calibration was greater than 10% for a full deployment (n=16), the deployment was divided into smaller 6-hour segments and the data-driven calibration procedure was performed within each segment (Table S1). In some cases (n=2), the deployment files were particularly noisy and, to preserve magnetometer data, those files were calibrated piecewise using smaller segment sizes (GHAL53 = 3hour segments, BBAL84 = 2hour segments).


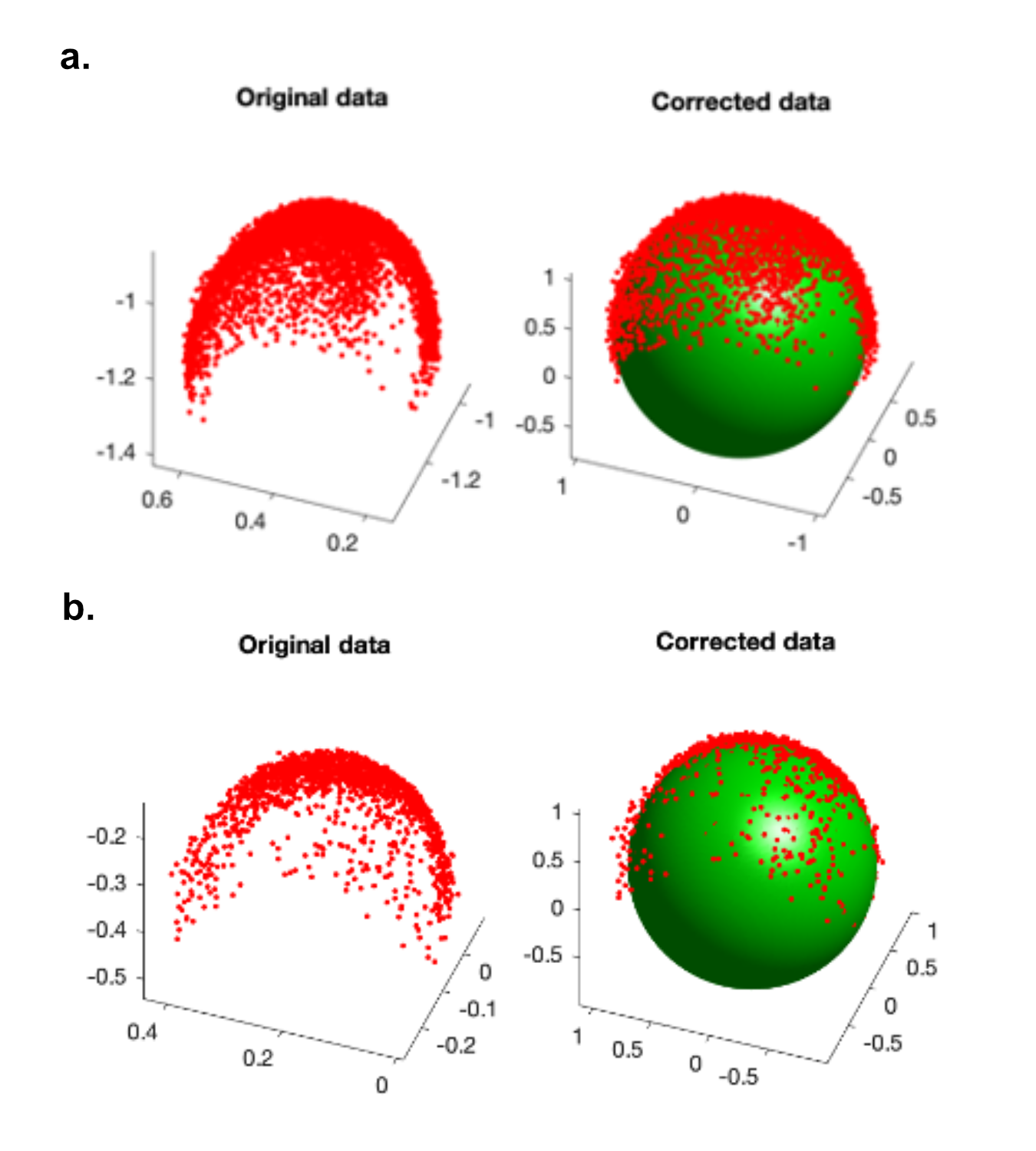


Supplemental Figure S1 Example spherical plots demonstrating the spherical fits of magnetometer data post-calibration. a) diagnostic plot for the calibration of the deployment for GHAL48, the single file calibration with the smallest residual error (0.026). b) diagnostic plot for a calibration performed within a 6-hour segment for BBAL20 with a residual error of 0.042.

Supplemental Table S1. Mean residual error (RE) for all magnetometer calibrations. Deployments with calibrations that resulted in > 10 % RE for the full deployment were subsequently divided into 6-hour segments, and calibrations were performed within each segment. Most segments resulted in calibrations with RE <10%, but two deployments (BBAL91 and BBAL84) had a mean RE much greater than 10% despite being calibrated piecewise within segments.

| Tag ID | No. Segments | No. Segments > 10% RE Threshold | Mean Residual Error across all segments |
| --- | --- | --- | --- |
| BBAL107 | 1 | 0 | 0.04390987 |
| BBAL108 | 1 | 0 | 0.06926963 |
| BBAL71 | 1 | 0 | 0.04382899 |
| BBAL86 | 1 | 0 | 0.05637026 |
| BBAL92 | 1 | 0 | 0.05715558 |
| GHAL26 | 1 | 0 | 0.09612711 |
| GHAL28 | 1 | 0 | 0.08080421 |
| GHAL47 | 1 | 0 | 0.03650931 |
| GHAL48 | 1 | 0 | 0.02579738 |
| GHAL65 | 1 | 0 | 0.03870720 |
| BBAL20 | 14 | 1 | 0.05344817 |
| BBAL21 | 33 | 1 | 0.04762794 |
| BBAL72 | 16 | 1 | 0.05156254 |
| GHAL51 | 33 | 1 | 0.04587050 |
| GHAL55 | 13 | 1 | 0.04348810 |
| BBAL100 | 26 | 2 | 0.05971629 |
| GHAL53 | 15 | 2 | 0.04572359 |
| GHAL58 | 20 | 3 | 0.06673888 |
| GHAL83 | 11 | 3 | 0.07038868 |
| BBAL62 | 16 | 4 | 0.07480086 |
| BBAL99 | 13 | 4 | 0.07710663 |
| BBAL91 | 21 | 5 | 0.57208563 |
| GHAL44 | 13 | 5 | 0.09448380 |
| GHAL70 | 8 | 5 | 0.13822381 |
| BBAL106 | 20 | 14 | 0.16603303 |
| BBAL84 | 82 | 36 | 0.29285704 |

Johnson, M. (2011). Measuring the orientation and movement of marine animals using inertial and magnetic sensors - a tutorial. In *Fine-scale animal movement workshop* (pp. 1–22). Hobart, Tasmania: Unpublished.
