# Supplementary material for: Hidden Markov models identify major movement modes in accelerometer and magnetometer data from four albatross species
Source: Mov Ecol. 2021 Feb 22;9:7. doi: 10.1186/s40462-021-00243-z (PMC7901071; doi:10.1186/s40462-021-00243-z)
Supplement: Supplementary file 2 — Additional file 2: Supplemental Table S2. Description of candidation features for the hidden Markov models. [file 40462_2021_243_MOESM2_ESM.docx]

**Additional File 2**

Supplemental Table S2: Candidate features for the hidden Markov models: The following eight features were summarized from 25 Hz accelerometer and magnetometer time series within consecutive fixed windows of 30-seconds. Features in bold were selected as input for the final HMM framework.

| **Candidate feature** | **IMU data stream** | **Method notes** | **Reason for candidacy** |
| --- | --- | --- | --- |
| 1. Dominant frequency   (‘df’) | Total heave acceleration | Identified using a fast-Fourier transform on the detrended timeseries. We used *findpeaks.m* in Matlab on *Frequency x Amplitude* plots to identify dominant frequencies in the signal. The ‘Dominant frequency’ was defined as the peak with maximum amplitude in the signal. | Flapping signals in bird flight show a high frequency and high amplitude in heave acceleration signals relative to flapping or soaring. |
| 1. **Highest frequency**   **(‘hf’)** | Total heave acceleration | Using the methods above, this feature was defined as the peak in the *Frequency x Amplitude* plot with the highest frequency. | Flapping flight is associated with high frequency acceleration and soaring flight with a lower frequency. |
| 1. Mean static heave   (‘ms’) | Static heave acceleration | Static acceleration was extracted from total acceleration using a 2-s running mean filter. | Static heave acceleration values were anticipated to have distributions that varied with bird behavior: while in dynamic soaring flight, birds showed the highest values of static heave acceleration. Conversely, when on the surface of the water, static acceleration approximated 1 G (the force of acceleration due to gravity) |
| 1. Standard deviation of static heave   (‘ss’) | Static heave acceleration | Static acceleration was extracted from total acceleration using a 2-s running mean filter. | Static heave acceleration showed very low deviation from the mean when birds were on the water and high deviation from the mean when the birds were soaring. |
| 1. **Top 5^th^ percentile of static heave acceleration**   **(‘p5’)** | Static heave acceleration | Static acceleration was extracted from total acceleration using a lowpass filter with a window of 2 seconds. | Static heave acceleration values were anticipated to have distributions that varied with bird behavior: while in dynamic soaring flight, birds showed the highest values of static heave acceleration. Conversely, when on the surface of the water, static acceleration approximated 1 G (the force of acceleration due to gravity) |
| 1. **Circular standard deviation**   **(‘sh’)** | Heading | Pitch and roll derived from accelerometer data were gimballed with triaxial magnetometer data to calculate heading (yaw). | We anticipated that the standard deviation of heading would be greatest while the birds were soaring and lowest while the birds were on water. |
| 1. Inter-quartile range of dynamic heave   (‘iqr’) | Dynamic heave acceleration | Dynamic acceleration was extracted by subtracting static from total acceleration. | We anticipated that the inter-quartile range of dynamic heave acceleration would be the greatest while the birds were flapping and the lowest while the birds were on the water. |
| 1. Mean Overall Dynamic Body Acceleration   (ODBA)  (‘mo’) | Dynamic acceleration, all axes. | ODBA was calculated by summing the absolute value of the dynamic acceleration across all three axes. | ODBA is a proxy of dynamic movement and energy expenditure had potential to reflect different activity levels associated with flapping (high energy), soaring (medium energy), and on water (low energy). |

Supplemental Table S3: Correlation Matrix of Candidate Features.

|  | ‘df’ | ‘hf’ | ‘ms’ | ‘ss’ | ‘p5’ | ‘sh’ | ‘iqr’ | ‘mo’ |
| --- | --- | --- | --- | --- | --- | --- | --- | --- |
| ‘df’ |  | 0.5 | -0.1 | -0.16 | -0.14 | -0.05 | 0 | 0.12 |
| ‘hf’ | 0.5 |  | -0.09 | -0.08 | -0.11 | 0.06 | 0.02 | 0.23 |
| ‘ms’ | -0.1 | -0.09 |  | 0.59 | 0.89 | 0.6 | 0.6 | 0.34 |
| ‘ss’ | -0.16 | -0.08 | 0.59 |  | 0.87 | 0.68 | 0.6 | 0.51 |
| ‘p5’ | -0.14 | -0.11 | 0.89 | 0.87 |  | 0.72 | 0.67 | 0.45 |
| ‘sh’ | -0.05 | 0.06 | 0.6 | 0.68 | 0.72 |  | 0.52 | 0.42 |
| ‘iqr’ | 0 | 0.02 | 0.6 | 0.6 | 0.67 | 0.52 |  | 0.83 |
| ‘mo’ | 0.12 | 0.23 | 0.34 | 0.51 | 0.45 | 0.42 | 0.83 |  |

**Reducing candidate features into a smaller set by inspecting their correlation matrix:**

- We evaluated the correlation among potential features to reduce the number of features used in the HMM. ‘df’ and ‘hf’ were moderately correlated with each other but had very low correlation values with the other candidate features, so we selected one of them. We chose **‘hf’** because we thought it would better distinguish flapping flight.
- ‘p5’ was highly correlated with both ‘ms’ (0.89) and ‘ss’ (0.87), so we selected ‘**p5**’ while setting aside ‘ms’ and ‘ss’.
- ‘**sh’** was moderately correlated (0.72) with ‘p5’ but we selected it regardless because we wanted to evaluate the contribution of it (as the only magnetometer-derived feature) to classification accuracy.
- ‘iqr’ was moderately to highly correlated with all features with the exception of ‘hf’ and ‘df’, so we did not pursue this feature.
- **‘mo’** was only mildly correlated with ‘p5’ (0.45) and ‘sh’ (0.42) and not at all with ‘hf’ (0.23), so it was also included in the first run of the HMM.
