# Supplementary material for: Hidden Markov models identify major movement modes in accelerometer and magnetometer data from four albatross species
Source: Mov Ecol. 2021 Feb 22;9:7. doi: 10.1186/s40462-021-00243-z (PMC7901071; doi:10.1186/s40462-021-00243-z)
Supplement: Supplementary file 3 — Additional file 3: Supplemental Figure S2. Feature distributions across species. [file 40462_2021_243_MOESM3_ESM.docx]

**Additional File 3**


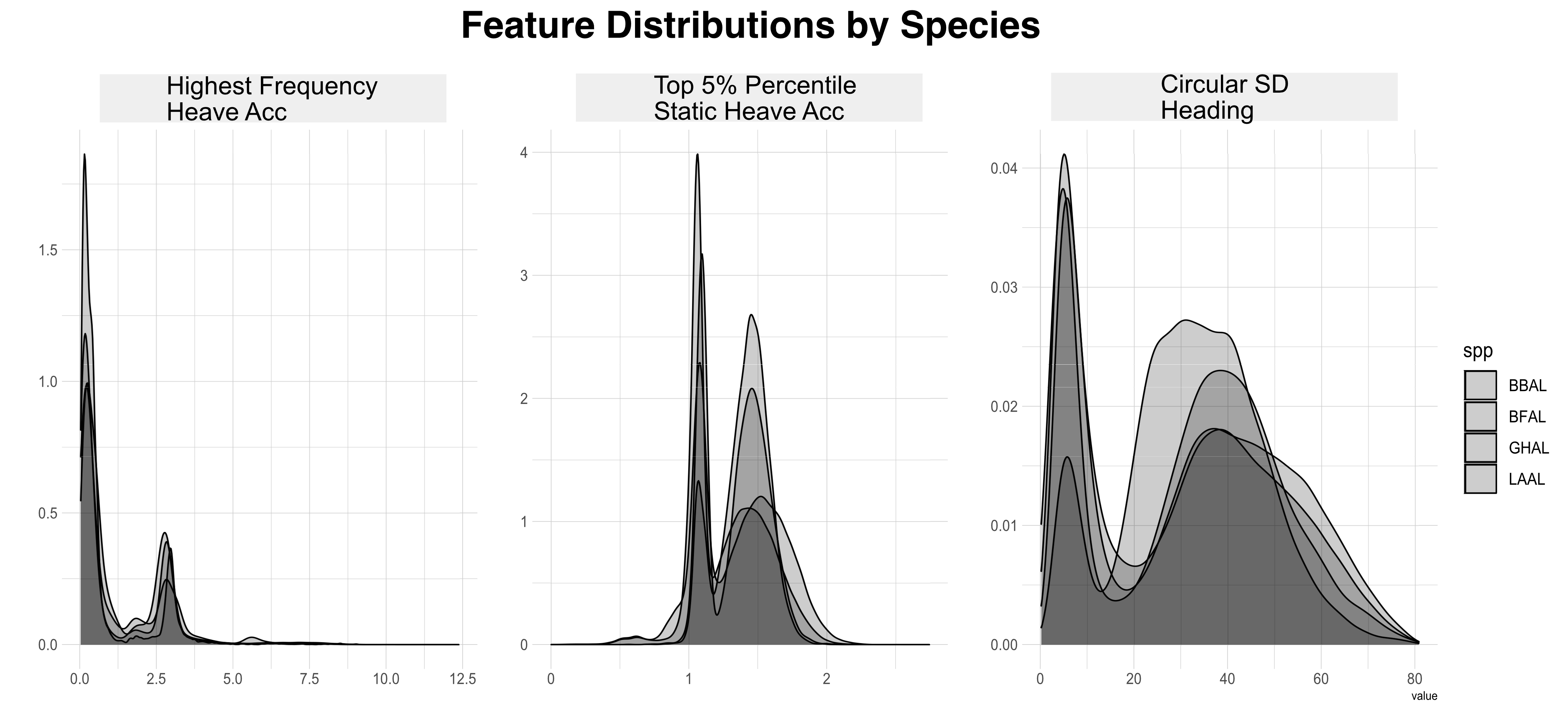


Supplemental Figure S2: Density histograms of the three final features with overlaying density curves for the four species in transparent grey. There is high overlap in feature distributions across species, indicating similar movement kinetics.
