# Supplementary material for: Hidden Markov models identify major movement modes in accelerometer and magnetometer data from four albatross species
Source: Mov Ecol. 2021 Feb 22;9:7. doi: 10.1186/s40462-021-00243-z (PMC7901071; doi:10.1186/s40462-021-00243-z)
Supplement: Supplementary file 4 — Additional file 4: Supplemental Table S3. HMM-converged parameters for the Weibull distribution of each feature and state. [file 40462_2021_243_MOESM4_ESM.docx]

**Additional File 4**

Supplemental Table S3. HMM-converged distribution parameters using the Weibull function for each model. State1 = Flapping Flight, State2 = Soaring Flight, State3 = On Water

| **Model-1: Accelerometer and Magnetometer Derived Features** | | | |
| --- | --- | --- | --- |
| ‘hf’ parameters | | | |
|  | State1 | State2 | State3 |
| Shape | 15.0888 | 0.7108 | 0.9905 |
| Scale | 2.9489 | 0.6260 | 1.0117 |
|  |  |  |  |
| ‘p5’ parameters | | | |
|  | State1 | State2 | State3 |
| Shape | 9.4104 | 7.7245 | 16.5623 |
| Scale | 1.4231 | 1.6133 | 1.0699 |
|  |  |  |  |
| ‘sh’ parameters | | | |
|  | State1 | State2 | State3 |
| Shape | 3.6206 | 3.6264 | 1.5436 |
| Scale | 48.5711 | 48.8939 | 9.2503 |

| **Model-2: Accelerometer Only Derived Features** | | | |
| --- | --- | --- | --- |
| ‘hf’ parameters | | | |
|  | State1 | State2 | State3 |
| Shape | 15.2449 | 0.7180 | 0.9811 |
| Scale | 2.9452 | 0.6136 | 1.0515 |
|  |  |  |  |
| ‘p5’ parameters | | | |
|  | State1 | State2 | State3 |
| Shape | 9.4167 | 7.1352 | 23.7176 |
| Scale | 1.4276 | 1.6036 | 1.0751 |
